# Comparative Efficacy of Bupivacaine Versus Ropivacaine for Postoperative Pain Control After Total Knee Arthroplasty: A Systematic Review and Meta-Analysis

**DOI:** 10.7759/cureus.99994

**Published:** 2025-12-24

**Authors:** Jake Belli, Philopateer Messeha, Grant Weiderman, Jacob Hostetter, Marcia Ballantyne

**Affiliations:** 1 Medicine, Lake Erie College of Osteopathic Medicine, Bradenton, USA; 2 Anesthesiology, Allegheny Health Network, Pittsburgh, USA; 3 Pathology, Lake Erie College of Osteopathic Medicine, Bradenton, USA

**Keywords:** bromage scale, bupivacaine, pain management, regional nerve block, rescue analgesia, ropivacaine, total knee arthroplasty, vas

## Abstract

Postoperative pain is a significant challenge that can delay early mobilization and prolong hospitalization after total knee arthroplasty (TKA). Effective pain management is critical to promote rehabilitation, reduce opioid consumption, and improve long-term patient outcomes. Bupivacaine and ropivacaine are widely utilized local anesthetics administered by way of peripheral nerve blocks, periarticular injections, and epidural anesthesia to help manage postoperative pain. This systematic review and meta-analysis aimed to compare postoperative pain levels after administering bupivacaine or ropivacaine via peripheral nerve blocks or periarticular injections. Secondary outcomes included length of stay (LOS), rescue analgesia, and motor blockade. Subgroup analysis was performed to differentiate between drug administration by way of peripheral nerve blocks and periarticular injections. A total of nine studies and 656 patients were included. There was no difference in pain levels at 6 hours (MD = 0.12, 95% CI, -0.41 to 0.65, P = 0.66), 12 hours (MD = - 0.01, 95% CI, -0.62 to 0.61, P = 0.98), and 24 hours postoperative (MD = 0.17; 95% CI, -0.27 to 0.61, P = 0.46) between peripheral nerve blocks and periarticular injections of bupivacaine and ropivacaine. At 72 hours postoperative, ropivacaine decreased pain levels when administered through peripheral nerve block only (MD = 0.82, 95% CI, 0.55 to 1.09, P = 0.002). There were no differences in LOS or rescue analgesia, but bupivacaine provided a greater degree of motor nerve blockade (MD = 0.18, 95% CI, 0.01 to 0.35, P = 0.04) assessed by the classical Bromage scale. The findings suggest that bupivacaine and ropivacaine provide comparable analgesic efficacy for postoperative pain control following TKA, indicating that the drug of choice may be due to secondary factors, including cost, availability, or provider preference.

## Introduction and background

Achieving adequate pain control while preserving muscle strength after total knee arthroplasty (TKA) has become an important objective in current anesthetic practice [1]. Despite major advances in surgical techniques, optimizing the anesthetic approach for TKA continues to pose a clinical challenge. Local morphine injections have been utilized post-TKA, but certain studies have found that the harmful side effects and insignificant effects on visual analogue scale (VAS) scores of pain do not make this drug an optimal choice [2]. Epidural analgesics have also been utilized due to lower postoperative nausea and vomiting, but the potential risk of epidural hematomas and hypotension does not currently make this the best candidate [3,4]. The development of multimodal analgesia, a method that utilizes multiple medications and modes of administration, has become the more common approach to treating pain preoperatively, intraoperatively, and postoperatively. A recent meta-analysis that looked at 106 articles about pain management in TKA concluded that the use of acetaminophen, nonsteroidal anti-inflammatory drugs (NSAIDs), cyclo-oxygenase (COX)-2 inhibitors, dexamethasone, adductor canal blocks in combination with local infiltrative analgesics (LIA), and periarticular injections is associated with more favorable pain outcomes [5]. In a network meta-analysis that compared 170 studies that considered peripheral nerve blocks, periarticular injections, epidural analgesics, and local anesthetics, pain reduction was the most significant when blocking multiple nerves at a time [6]. However, the varying protocols for analgesic use as well as the varying intensity of postoperative pain highlight the need for a more universal approach.

Ropivacaine and bupivacaine are amide local anesthetics whose mechanism of action is to block nerve conduction by binding to sodium channels [7]. Bupivacaine was first synthesized in 1957 and introduced into clinical practice in the early 1960s, where it became widely adopted for neuraxial and peripheral regional anesthesia due to its prolonged nerve blockade [7]. However, the concerns of dose-related cardiotoxicity associated with bupivacaine prompted the development of newer anesthetic agents such as ropivacaine [8]. Ropivacaine is less lipophilic than bupivacaine and is less likely to penetrate large myelinated fibers, thus decreasing motor blockade, which is favorable to facilitate early rehabilitation and mobilization after surgery [9]. Ropivacaine is also associated with a reduced potential for cardiovascular and central nervous system toxicity [8]. While toxicity plays a role in the anesthetic agent of choice, financial considerations may also impact the drug of choice. At Mount Sinai Hospital in New York City, a 20 mL vial of 0.2% ropivacaine costs $4.29, whereas a 30 mL vial of 0.25% bupivacaine costs $1 [10]. Ropivacaine is approximately ten times more costly per milligram, which may influence anesthetic selection across institutions. 

Many studies have inconsistent and conflicting data regarding pain outcomes when comparing bupivacaine to ropivacaine [11-19]. To address this uncertainty, a comprehensive synthesis of the literature is required. In assessing analgesic efficacy, visual analogue scale (VAS) scores at 24 hours have emerged as a clinically meaningful value to compare standardized pain levels among various fields. The central objective of this systematic review and meta-analysis was to compare bupivacaine to ropivacaine in patients undergoing TKA to determine if VAS scores differed significantly at 24 hours. The pooled analysis of pain outcomes, length of stay, motor blockade, and opioid consumption will offer insight into recent TKA studies, which have the potential to reform anesthetic practice and improve patient outcomes.

## Review

Methods

Literature Search and Selection

This systematic review and meta-analysis were conducted following the Preferred Reporting Items for Systematic Review and Meta-Analyses (PRISMA) 2020 [20] guideline and Cochrane [21] checklist to complete the systematic review and meta-analysis. Online databases were searched for articles, including PubMed, Scopus, Cochrane Library, and Google Scholar. The keywords applied for the search were “bupivacaine,” “ropivacaine,” “VAS,” “total knee arthroplasty,” and “pain management.” Three reviewers performed study screening independently and in duplicate (JB, PM, GW). All articles were assessed to determine if they fit the inclusion criteria described below, and relevant data were extracted in the data sheets. Any disagreements were resolved through discussion until a consensus was reached.

Eligibility Criteria

The inclusion criteria consisted of randomized controlled trials (RCTs), cohort studies, and comparative observational studies that compared the use of bupivacaine versus ropivacaine as a local anesthetic in TKA. The method of injections included in the study was peripheral nerve blocks and periarticular injections. We included cocktail mixtures in which bupivacaine or ropivacaine was mixed with other drugs [22]. Each article was required to report the VAS score 24 hours after the TKA procedure, with this designated as the primary outcome of this study. Secondary outcomes included the percentage of patients receiving rescue analgesia, LOS in hospital after the procedure, the level of motor block as defined by the Bromage scale, and VAS scores at 6 hours, 12 hours, and 72 hours after TKA [23]. We excluded review articles, articles published before 2010, those that used epidural administration, those that did not directly compare a bupivacaine group versus a ropivacaine group, those that used other drugs like ketamine as the control, and articles that did not have accessible full texts available.

Statistical Analysis

**Figure 1:**
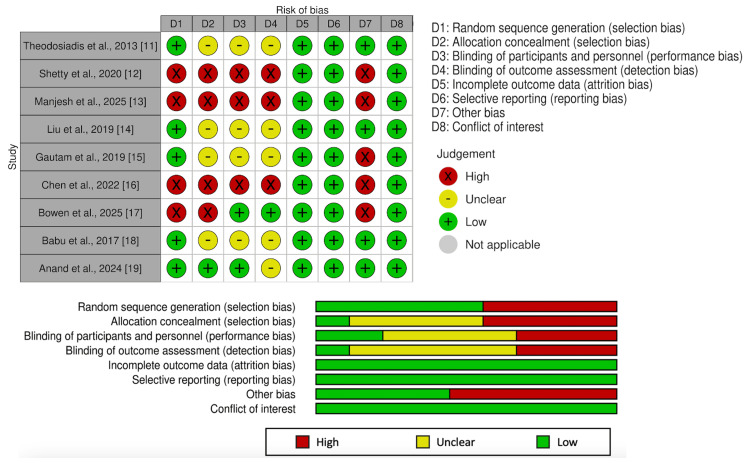
Summary and graph showing the risk and bias in the included studies.

Random-effects meta-analyses were conducted using RStudio (version 4.3.0, 2023; Posit, Boston, MA) [24]. Subgroup analysis was performed with peripheral nerve blocks and periarticular injections due to the difference in injection location. Clinical heterogeneity was expected across studies given variation in age, comorbid conditions, and perioperative characteristics, which could influence postoperative pain outcomes [25]. This variability calls for the use of the random-effects model to minimize the risk of false-positive findings [26]. An I^2^ statistic value of > 50% indicated significant heterogeneity amongst studies [27]. Pooled mean differences (MDs) were calculated for each outcome with 95% confidence intervals (CI). The results of the meta-analysis were displayed as forest plots. The risk of bias associated with the selected studies was assessed using the Cochrane risk of bias (RoB) tool for randomized trials RoB1 (Figure 1) [28]. When data was only presented in graphical form, WebPlot Digitizer was used to estimate the numerical value [29]. Funnel plots were not conducted due to the number of studies analyzed [30].

Results

Screening

**Figure 2:**
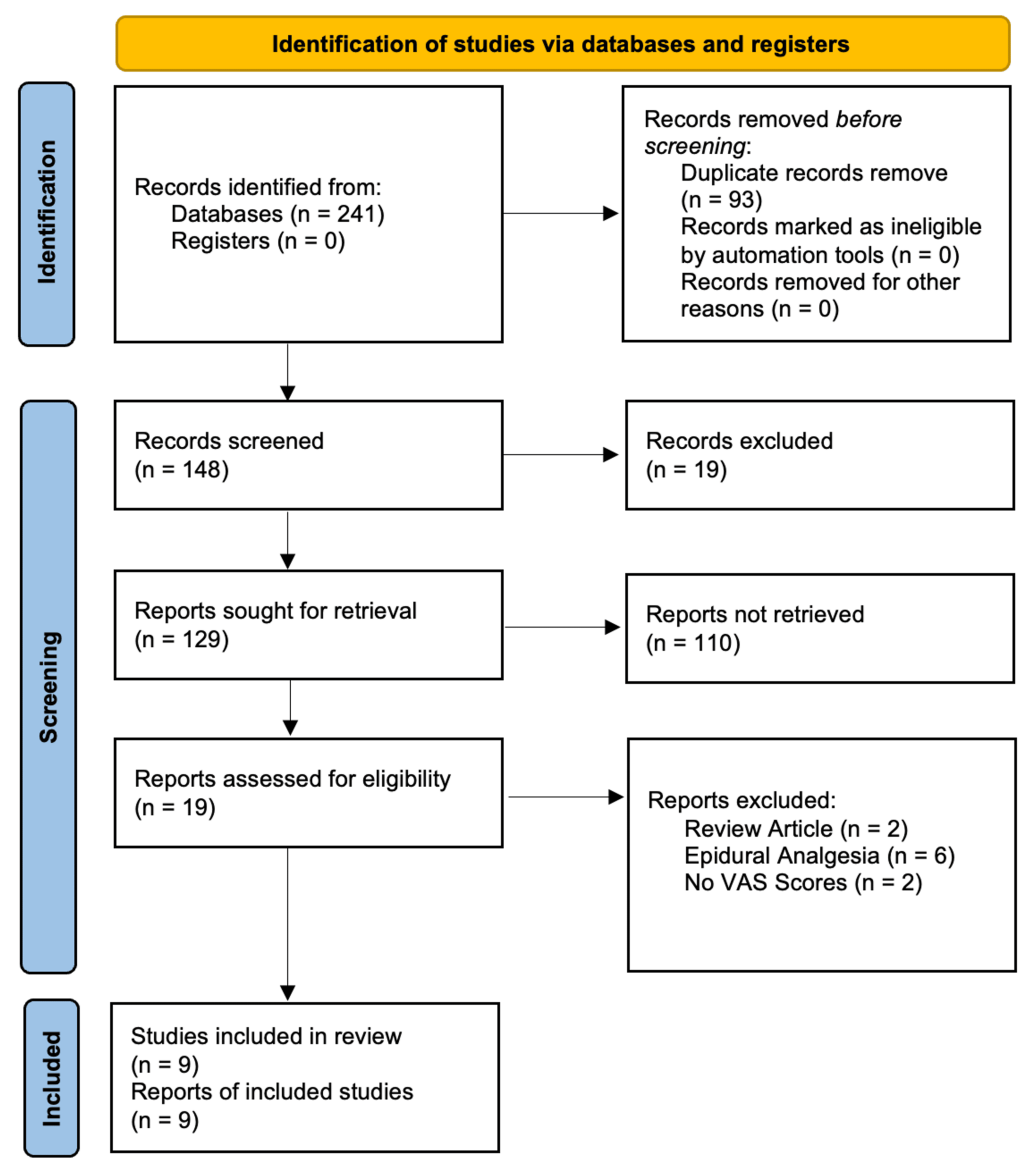
PRISMA flow diagram. PRISMA: Preferred Reporting Items for Systematic Reviews and Meta-Analyses; VAS: Visual Analogue Scale.

A total of 241 articles were identified from the four databases that were searched. After removing duplicates, 148 articles were left for screening. After reviewing the titles and abstracts of the 148 articles that remained, 129 of the articles were excluded because they failed to directly compare pain levels 24 hours after TKA following the administration of bupivacaine versus ropivacaine. Of the remaining 19 full-text articles assessed for eligibility, 10 were excluded because they were review articles, did not report VAS scores, or involved epidural administration. The remaining nine articles were fully relevant to the research topic and were considered in the systematic review (Table 1) and meta-analysis. Figure 2 shows the PRISMA (Preferred Reporting Items for Systematic Reviews and Meta-Analyses) 2020 guidelines that were followed during the systematic review.

**Table 1:** Systematic review of articles analyzed. VAS: Visual Analogue Scale; RCT: randomized controlled trial.

Authors	Study design	Procedure	Bupivacaine dosage	Ropivacaine dosage	Cocktail mixture with bupivacaine	Cocktail mixture with ropivacaine	Main findings
Theodosiadis et al. (2013) [11]	RCT	Femoral, lateral femoral cutaneous, and obturator nerve block	0.50%	0.50%	None	None	No difference in VAS scores at 24 hours.
Shetty et al. (2020) [12]	RCT	Periarticular injection	0.50%	0.50%	Tranexamic acid, butorphanol, ketorolac, tramadol, antibiotic	Tranexamic acid, butorphanol, ketorolac, tramadol, antibiotic	Ropivacaine decreases VAS scores at 24 hours and has better postoperative knee range of motion.
Manjesh et al. (2025) [13]	Comparative observational	Femoral nerve block	0.125 %	0.10%	Dexmedetomidine	Dexmedetomidine	No difference in VAS scores at 24 hours. No difference in nausea and vomiting.
Liu et al. (2019) [14]	RCT	Periarticular injection	0.50%	1.00%	Methylprednisolone, adrenaline, morphine, normal saline	Ketorolac, adrenaline, morphine, normal saline	Ropivacaine decreases VAS scores at 24 hours.
Gautam et al. (2019) [15]	Prospective comparative	Periarticular injection	0.25%	0.75%	Methylprednisolone, Fentanyl, Cefuroxime, Normal saline	Epinephrine, Clonidine, Cefuroxime, Normal saline	Bupivacaine decreases VAS scores at 24 hours. By 72 hours, the treatments had no difference in VAS scores.
Chen et al. (2022) [16]	Retrospective cohort	Adductor canal nerve block	0.25%	0.50%	Liposomal bupivacaine	None	Mean opioid consumption was lower in the liposomal bupivacaine group at 8 and 24 hours. At 48 hours, ropivacaine had lower VAS scores.
Bowen et al. (2025) [17]	RCT	Periarticular injection	0.50%	0.50%	Liposomal bupivacaine, epinephrine, clonidine, normal saline, ketorolac	Epinephrine, clonidine, normal saline, ketorolac	No difference in VAS scores at 24 hours. No difference in opioid consumption.
Babu et al. (2017) [18]	Prospective cohort	Femoral nerve block	0.13%	0.20%	None	None	No difference in VAS scores at 24 hours. No difference in opioid consumption. Bupivacaine provided better analgesic effects and better motor blockade.
Anand et al. (2024) [19]	RCT	Femoral, lateral femoral cutaneous, and obturator nerve block	0.25%	0.25%	None	None	Ropivacaine decreases VAS scores at 24 hours with greater duration of analgesic effects. Ropivacaine used less rescue analgesics

Pain Scores

**Figure 3:**
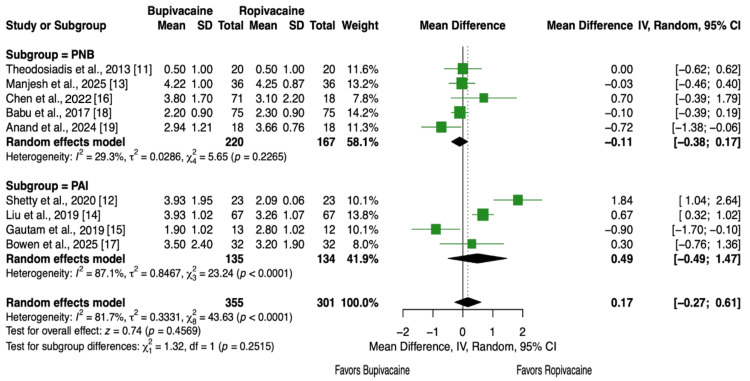
Forest plot showing pain scores (VAS) at 24 hours postoperative at rest. PNB: peripheral nerve block; PAI: periarticular injection.

Nine articles and 656 patients [11-19] reported VAS scores at 24 hours post-TKA. There were no statistically significant differences in pain levels between patients who received bupivacaine versus patients who received ropivacaine for TKA (MD = 0.17; 95% CI, -0.27 to 0.61, P = 0.46, Figure 3). To account for differences in anesthetic administration, subgroup analysis between peripheral nerve block (MD = -0.11, 95% CI, -0.38 to 0.17, P = 0.23, Figure 3) and periarticular injection (MD = 0.49, 95% CI, -0.49 to 1.47, P > 0.05, Figure 3) yielded no statistically significant difference in pain levels after 24 hours.

**Figure 4:**
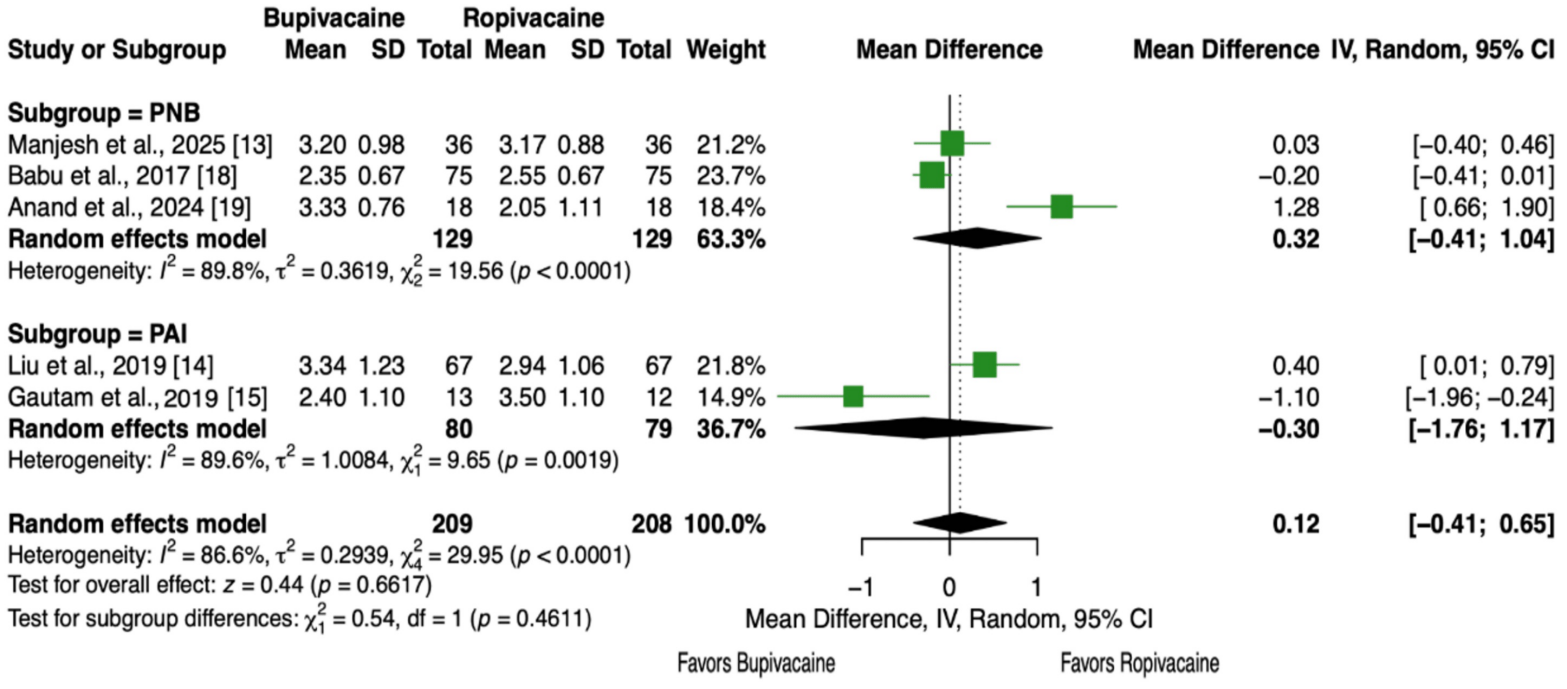
Forest plot showing pain scores (VAS) at 6 hours postoperative at rest.

Five articles and 417 patients [13-15,18,19] reported VAS scores at 6 hours. There were no statistically significant differences in pain levels between bupivacaine and ropivacaine (MD = 0.12, 95% CI, -0.41 to 0.65, P = 0.66, Figure 4), and this finding was consistent when analyzing subgroups (P = 0.46, Figure 4).

**Figure 5:**
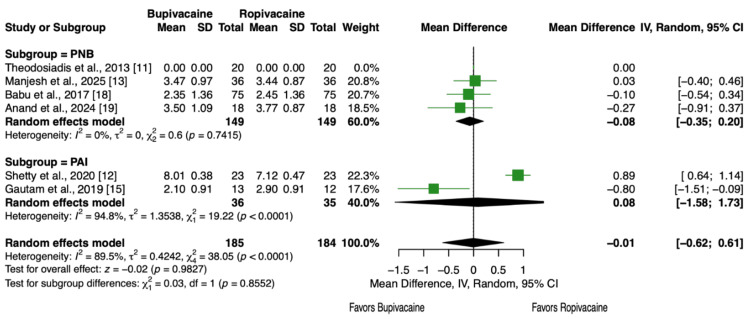
Forest plot showing pain scores (VAS) at 12 hours postoperative at rest.

Six articles and 369 patients [11-13,15,18,19] reported VAS scores at 12 hours. No statistically significant difference in pain levels was observed between the two groups (MD = - 0.01, 95% CI, -0.62 to 0.61, P = 0.98, Figure 5). This result remained consistent across the peripheral nerve block (PNB) and periarticular injection (PAI) subgroups (P = 0.86, Figure 5).

**Figure 6:**
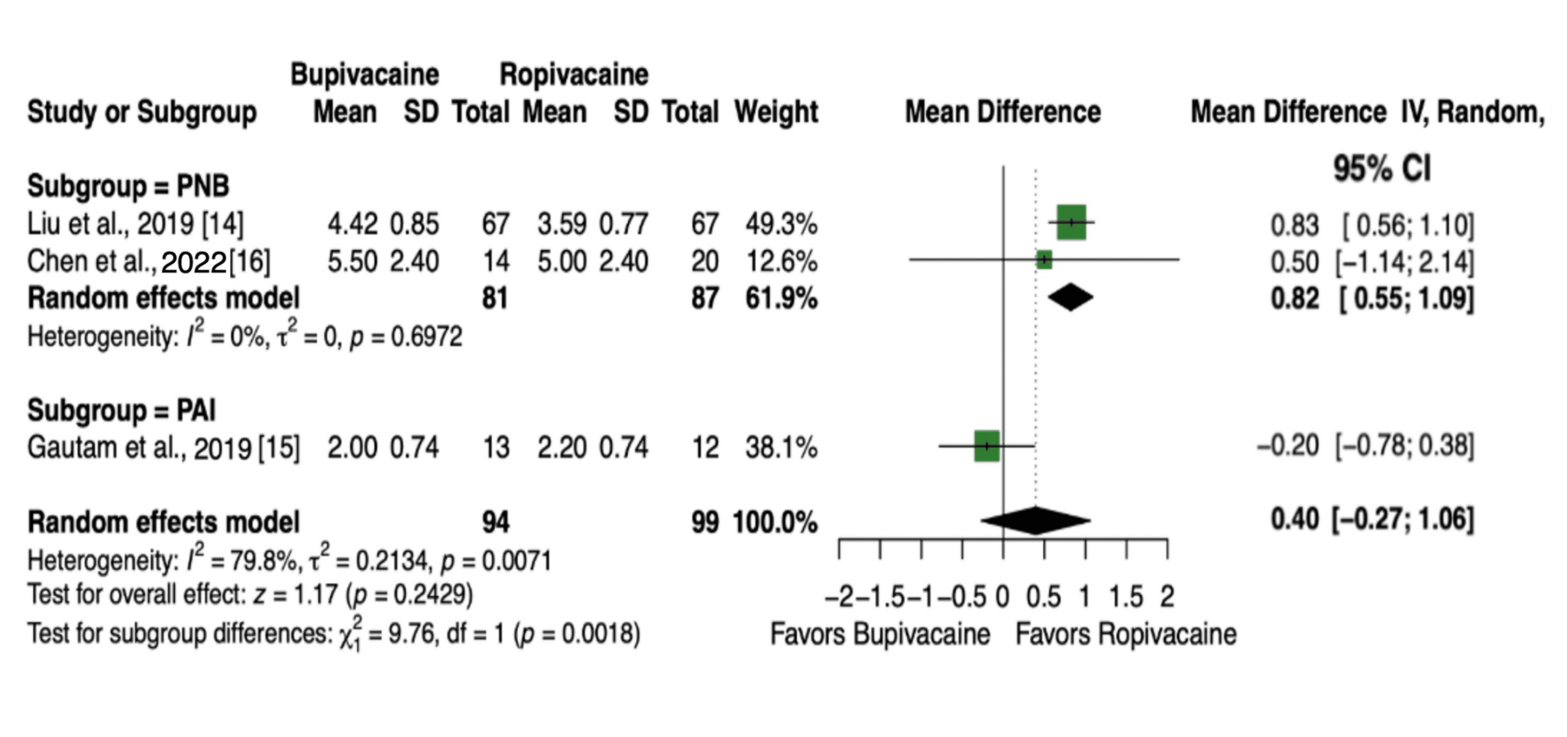
Forest plot showing pain scores (VAS) at 72 hours postoperative at rest.

Three articles and 193 patients [14-16] reported VAS scores at 72 hours postoperatively. No statistically significant differences in pain levels were observed when comparing the overall effect (MD = 0.40, 95% CI, -0.27 to 1.06, P = 0.24, Figure 6), but there was a statistically significant reduction in pain when using ropivacaine in the PNB subgroup at 72 hours postoperative (MD = 0.82, 95% CI, 0.55 to 1.09, P = 0.002, Figure 6).

Secondary Outcomes

**Figure 7:**
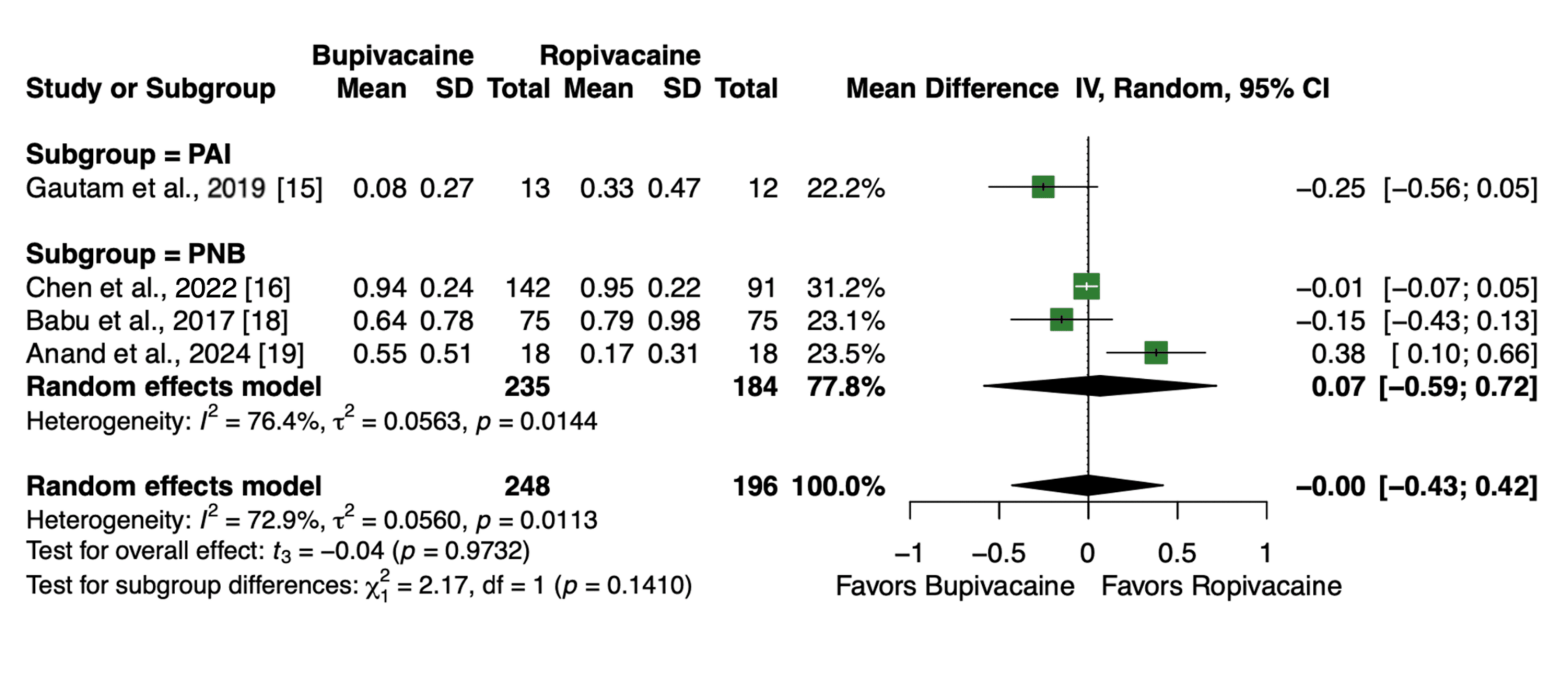
Forest plot showing the percentage of patients who received rescue analgesia within 24 hours after operation.

Four articles and 444 patients [15,16,18,19] reported the percentage of patients who received rescue analgesia, such as fentanyl or morphine, within 24 hours of the operation. There was no statistically significant difference in the percentage of patients who received rescue analgesia after receiving bupivacaine or ropivacaine post-TKA, regardless of the route of administration of the anesthetic during the procedure (MD = 0.00, 95% CI, -0.43 to 0.42, P = 0.97, Figure 7).

**Figure 8:**
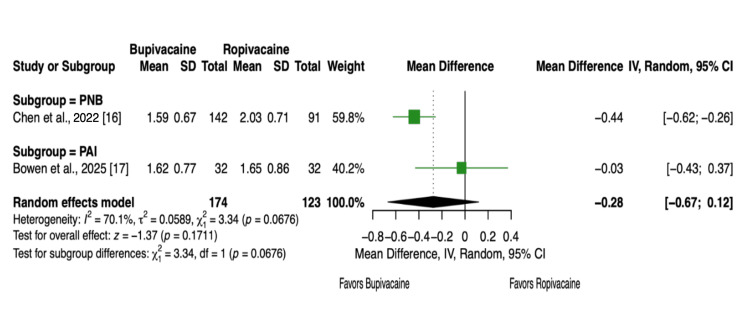
Forest plot showing the length of stay (LOS) in the hospital after the procedure.

Two articles and 297 patients [16,17] reported LOS in the hospital after the procedure. LOS did not appear to correlate with the use of bupivacaine or ropivacaine (MD = -0.28, 95% CI, -0.67 to 0.12, P = 0.17, Figure 8) and most likely depended on factors such as age, comorbidities, and preoperative health [25].

**Figure 9:**
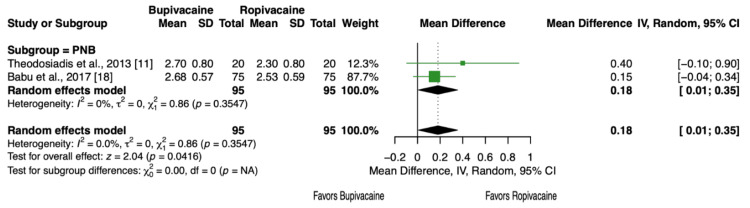
Forest plot showing the degree of nerve block assessed by the Bromage scale.

Two articles and 190 patients [11,18] reported the Bromage scale values, which assess the degree of nerve block after administration of an anesthetic [23] (Table 2). Each study reported a higher mean Bromage scale score with bupivacaine, indicating a greater degree of nerve block, and this difference was statistically significant (MD = 0.18, 95% CI, 0.01 to 0.35, P = 0.04, Figure 9).

**Table 2:** Bromage scale for motor nerve block. Adapted with permission from [31].

Grade	Criteria	Degree of block
I	Free movement of legs and feet	0%
II	Can flex knees, with free movement of feet	33%
III	Unable to flex knees, but with free movement of feet	66%
IV	Unable to move legs or feet	100%

Discussion

Pain is the most common symptom following surgery. Insufficient pain control during the first 24 hours can hinder rehabilitation, which emphasizes the importance of facilitating ambulation to optimize outcomes [32]. Across 656 patients from nine trials, no significant difference in VAS scores was observed at 24 hours (Figure 3). Our findings support the results of Theodosiadis et al., Manjesh et al., Chen et al., Bowen et al., and Babu et al., who all concluded no difference in VAS scores at 24 hours [11,13,16-18]. While Shetty et al., Liu et al., and Anand et al. [12,14,19] reported lower VAS scores at 24 hours with ropivacaine, and Gautam et al. [15] found lower scores with bupivacaine, neither of these outcomes was seen in this meta-analysis. These results suggest that providers should not base the choice between bupivacaine and ropivacaine solely on analgesic efficacy, as both agents demonstrated comparable effects on pain at this time point. Such discrepancies may reflect differences in dosing concentration, different formulation, or route of administration. Additional comparisons at 6, 12, and 72 hours postoperatively (Figures 4-6) similarly showed no significant differences at 6 or 12 hours; however, at 72 hours, patients receiving ropivacaine via peripheral nerve block reported significantly lower pain scores compared with those receiving bupivacaine. More high-quality studies are needed to improve the certainty of these findings.

Opioids remain an important component of postoperative pain management. However, minimizing their use is critical due to the well-recognized adverse effects, including nausea, somnolence, constipation, and the risk of dependence [33]. In this meta-analysis, four included studies assessed the need for rescue opioid analgesia, which is defined as the administration of fentanyl or morphine within the first 24 hours following surgery. Across a combined 444 patients, no statistically significant difference was observed between the bupivacaine and ropivacaine groups for rescue opioid requirements (Figure 7). This finding further supports the conclusion that both agents provide comparable analgesic efficacy in the immediate postoperative period. The lack of difference in opioid consumption is clinically relevant, as it suggests that the choice of local anesthetic does not significantly influence reliance on opioids for breakthrough pain in the first 24 hours after TKA. Our findings at 24 hours are consistent with the findings in Chen et al., but this study finds that liposomal bupivacaine and bupivacaine mixture decreases morphine use at 8 hours postoperative [16]. This finding in Chen et al. highlights two limitations of our study: the lack of analysis at different time periods for rescue analgesia and the inability to subanalyze liposomal bupivacaine and bupivacaine as two separate anesthetics [16]. However, Ji et al. found that liposomal bupivacaine did not show a reduction in opioid consumption in 86% of the randomized controlled trials analyzed [34]. Given the increasing emphasis on multimodal analgesia and opioid-sparing strategies, these results highlight that either agent can be appropriately incorporated into postoperative protocols without altering opioid requirements.

Another important outcome evaluated was motor blockade, assessed using the Bromage scale. This meta-analysis found that bupivacaine was associated with significantly higher Bromage scores, indicating denser motor blockade (Figure 9). This finding supports the idea that ropivacaine is less likely to penetrate larger motor fibers, such as those of the quadriceps, resulting in less motor impairment [8]. The lower Bromage scores observed with ropivacaine suggest that it may be more favorable for early ambulation following TKA. Supporting this, Shetty et al. reported greater mean knee flexion in the ropivacaine group at both 12 hours (39° vs 32°) and 24 hours (93° vs 79°) postoperatively [12]. Liu et al. further demonstrated that patients receiving ropivacaine achieved better knee range of motion at 3, 7, 10, and 14 days postoperatively, and more often achieved a good or excellent rehabilitation outcome at day 14 as measured by the Hospital for Special Surgery (HSS) scale [14]. The HSS scale incorporates pain, function, range of motion, strength, deformity, instability, and need for assistive devices, underscoring the multifaceted benefits of improved early mobility. Similarly, Theodosiadis et al. and Babu et al. also reported lower motor blockade with ropivacaine compared to bupivacaine [11,18]. However, Gautam et al. found no difference in knee flexion between groups at multiple postoperative time points (3, 7, 9, 11, 13, 21, and 42 days), although they did observe less extension lag with bupivacaine at 24 hours and 5 days [15]. Improved range of motion at these early time points is essential to facilitate earlier ambulation and rehabilitation, which in turn has been associated with shorter hospital stays and fewer physical therapy sessions required to restore gait and balance [35]. Despite differences in motor blockade, there was no difference in LOS between bupivacaine and ropivacaine. However, these discrepancies highlight the need for further investigation into how local anesthetic choice affects long-term functional recovery.

The clinical relevance of these findings extends beyond rehabilitation. Early ambulation is also a key factor in reducing the risk of venous thromboembolism (VTE), one of the most serious complications following TKA. A nationwide Korean claims study by Lee et al. demonstrated that VTE incidence was highest in the immediate postoperative period, particularly among elderly patients and those with delayed mobilization [36]. Similarly, Postajian et al. emphasizes that postoperative immobility following TKA contributes to venous stasis, a central component of Virchow’s triad, thereby increasing the risk of deep vein thrombosis and subsequent pulmonary embolism [37]. Thus, anesthetic choice may indirectly influence thromboembolic risk by determining how quickly patients regain quadriceps function and ambulate. By limiting motor blockade, ropivacaine may not only facilitate earlier functional recovery but also play an important role in reducing VTE incidence after TKA.

Limitations

This study has several limitations that should be acknowledged. First, our study disregarded any article that did not have full-text access or was published in a different language. Second, there was heterogeneity in terms of anesthetic techniques, drug concentrations, and the use of adjunct drugs, which had the potential to influence pain outcomes. Liposomal bupivacaine is a long-lasting local anesthetic formulation that may provide clinically different pain outcomes than bupivacaine. However, subgroup analysis was not performed due to the lack of studies that included the liposomal formulation. Several studies used cocktail mixtures in which bupivacaine or ropivacaine was combined with other agents. The inability to definitively assess whether bupivacaine or ropivacaine was affecting outcomes versus other agents in the cocktail mixture introduces confounding bias and limits the ability to compare the efficacy of the two directly. A further limitation relates to the subgroup analysis of peripheral nerve block and periarticular injection. The number of studies within each subgroup was small, which limited the ability to generate strong conclusions.

## Conclusions

Our meta-analysis shows evidence that postoperative pain at 24 hours following TKA is comparable between bupivacaine and ropivacaine. While certain studies have reported various findings favoring one agent over the other, the pooled evidence of VAS scores suggests that both anesthetics provide similar analgesia. Ropivacaine was shown to reduce pain levels at 72 hours in the peripheral nerve block subgroup, but the limited number of studies diminishes the strength of this finding. Given their similar efficacy, the choice of anesthetic agent is often guided by cost, safety profiles, and provider preference. Ropivacaine is considerably more expensive yet does not offer clear analgesic benefits over bupivacaine, thus inclining institutions to opt for the more cost-effective option. However, the drawback of bupivacaine is increased motor blockade, which can halt early remobilization following TKA, an important factor that is associated with improved patient outcomes.
